# Inter-individual responses of post-exercise hypotension in older adults with hypertension: An exploratory analysis of different exercise modalities

**DOI:** 10.3389/fphys.2022.1050609

**Published:** 2022-11-23

**Authors:** Leandro O. Carpes, Lucas B. Domingues, Otávio Bertoletti, Sandra C. Fuchs, Rodrigo Ferrari

**Affiliations:** ^1^ Postgraduate Program in Cardiology, School of Medicine, Universidade Federal do Rio Grande do Sul, Porto Alegre, Brazil; ^2^ Sports and Exercise Training Study Group, Hospital de Clínicas de Porto Alegre, Porto Alegre, Brazil; ^3^ Postgraduate Program in Epidemiology, School of Medicine, Universidade Federal do Rio Grande do Sul, Porto Alegre, Brazil

**Keywords:** endurance exercise, strength exercise, post-exercise hypotension, high blood pressure, physical exercise

## Abstract

**Background:** Various physical exercise modalities can acutely reduce blood pressure (BP). However, not all individuals respond similarly after an exercise session.

**Purpose:** To measure inter-individual variations in 24-h BP after a single bout of various exercise modalities in older adults with hypertension.

**Methods:** This retrospective study analyzed data from participants with hypertension (≥60 years) previously included in three randomized controlled trials on this topic. BP was assessed using ambulatory BP monitoring. We compared the mean changes in total 24-h, daytime, and nighttime BP after aerobic (AE, *n* = 19), combined (COMB, *n* = 19), resistance (RES, *n* = 23), and isometric handgrip (ISO, *n* = 18) exercise sessions to a non-exercising control session (C). The minimum detectable changes to classify the participant as a “Responder” for the corresponding exercise protocol were 4 and 2 mmHg for systolic and diastolic BP, respectively.

**Results:** The prevalence of Responders for systolic BP was as follows: AE 24-h: 37%, daytime: 47% and nighttime: 37%; COMB 24-h: 26%, daytime: 21% and nighttime: 32%; RES 24-h: 26%, daytime: 26% and nighttime: 35%; and ISO 24-h: 22%, daytime: 22% and nighttime: 39%. For diastolic BP, the prevalence of Responders was as follows: AE 24-h: 53%, daytime: 53% and nighttime: 31%; COMB 24-h: 26%, daytime: 26% and nighttime: 31%; RES 24-h: 35%, daytime: 22% and nighttime: 52%; and ISO 24-h: 44%, daytime: 33% and nighttime: 33%.

**Conclusion:** There was a high inter-individual variation of BP after a single bout of various exercises in older adults. Responders had higher BP values on the control day without exercise. Various exercise modalities might acutely reduce 24-h BP in older adults with hypertension.

## 1 Introduction

Hypertension is a major modifiable risk factor for cardiovascular disease; its prevalence increases over a lifespan (Fuchs and Whelton, 2020). It is estimated that 7 out of 10 adults aged 65 years and older have been diagnosed with high blood pressure (BP) (Muli et al., 2020; NCD Risk Factor Collaboration, 2021). Physical exercise is essential for hypertension treatment; it reduces BP and slows the progression of cardiovascular diseases among adults with hypertension (Pescatello et al., 2019). Moderate-intensity continuous aerobic training (Cornelissen and Smart, 2013), high intensity interval training (Carpes et al., 2022), and combined training (Álvarez et al., 2022) have been described as effective exercise strategies to reduce BP.

The chronic hypotensive effect of physical training appears to be related to the sum of the acute BP reduction that occurs after an exercise session (a phenomenon termed “post-exercise hypotension”) (Kenney and Seals, 1993). This acute effect might predict BP reduction after chronic training interventions (Liu et al., 2012), which can be detected compared to resting values or a typical day without exercise. The magnitude of BP reduction might be sustained over a prolonged period, during activities of daily living and sleeping as assessed by ambulatory BP monitoring (ABPM) (Bliziotis et al., 2012). The nighttime BP assessment after exercise is particularly important because nighttime BP is a stronger predictor of all-cause mortality and cardiovascular events than daytime BP in patients with hypertension (Fagard et al., 2008; Hansen et al., 2011).

The acute reduction on 24-h BP after exercise might vary according to different exercise modalities (aerobic exercise: −2.7/−1.3 mmHg, resistance exercise: 0.3/−0.8 mmHg, and combined: 0.5/1 mmHg for systolic and diastolic BP, respectively) (Saco-Ledo et al., 2021). Our research group has investigated post-exercise hypotension after aerobic, isometric, dynamic resistance exercises, and combined aerobic and resistance exercises in older adults with hypertension (Ferrari et al., 2017; Machado Filho et al., 2020; Schimitt et al., 2020; Carpes et al., 2021; Domingues et al., 2021; Bertoletti et al., 2022); these trials represent the average effect for group behavior. However, there is potential for inter-individual variability in BP responses (Bouchard and Rankinen, 2001), which could be further explored based on whether individuals were classified as Responders (i.e., BP decreases after exercise) or Non-responders (BP does not change or increases after exercise) (Lima et al., 2015; Álvarez et al., 2018).

Because it is essential to evaluate inter-individual 24-h BP responses after a bout of various exercise modalities in older adults, we performed an exploratory analysis of pooled individual participant data of randomized clinical trials, aiming to classify the Responders with post-exercise hypotension after aerobic, combined, isometric, and dynamic resistance exercises.

## 2 Materials and methods

### 2.1 Study design and participants

This is a post hoc analysis of pooled randomized clinical trials (RCTs) from three previously published RCTs (Ferrari et al., 2017; Schimitt et al., 2020; Bertoletti et al., 2022). These primary studies evaluated acute BP responses measured by ABPM after exercise and non-exercising control sessions. The inclusion criteria were older adults (≥60 years) (Mathers et al., 2015) with previously diagnosed hypertension by a physician and not engaged in structured exercise programs in the last 3 months prior to the start of the trial. Exclusion criteria included the previous diagnosis of heart failure, current smoking, musculoskeletal problems that restrained subjects from exercising, changes in antihypertensive medications throughout the trial, and participation in structured exercise programs in the previous 3 months.

Two studies were crossover RCTs (Ferrari et al., 2017; Schimitt et al., 2020) and one was a parallel RCT (Bertoletti et al., 2022), in which we extracted the resting and handgrip data from the same participant to maintain homogeneity in the comparisons within groups during the analysis of this exploratory study.

### 2.2 Characteristics of the experimental sessions

All experimental sessions (exercise vs. control) were performed at the same time of day (Ferrari et al., 2017; Bertoletti et al., 2022 in the morning; and Schimitt et al., 2020 in the afternoon) to control for potential diurnal variation in BP and residual effects of BP-lowering medications. Before the sessions, participants were instructed to avoid physical exercise for 24 h and throughout the study. They were asked to maintain their usual diet, avoid alcohol, coffee, and other stimulant substances, and not drink water during the experimental sessions. Participants taking BP-lowering medications were requested to maintain their current treatment throughout the investigation.

**FIGURE 1:**
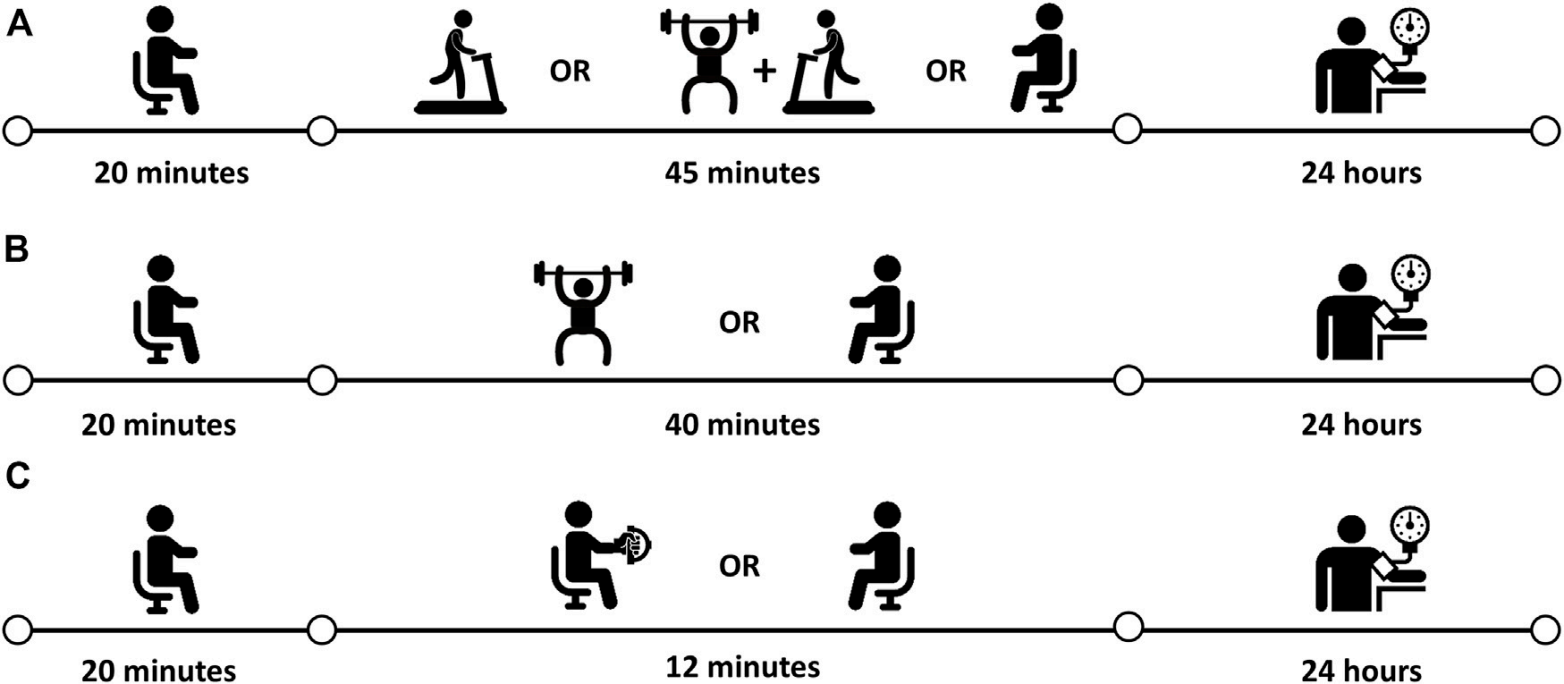
Experimental design and protocol. **(A)** Ferrari et al. (2017)–aerobic, combined e control sessions; **(B)** Schimitt et al. (2020)–resistance and control sessions; **(C)** Bertoletti et al. (2022)–isometric handgrip and control sessions.

Each session comprised 20 min of rest before and 60 min of rest after exercise or control. Afterward, participants underwent the 24-h ABPM (Spacelabs model 90207, Spacelabs Healthcare, Snoqualmie, United States) programmed to obtain BP measurements every 15 min during the day and every 20 min at night (O’Brien et al., 2013). The daytime period started immediately after the experimental sessions, the nighttime period started between 10 and 11 p.m. for 8 h, and the second daytime started between 6 and 7 a.m. until the time that the ABPM was placed on the previous day, completing the 24 h. The mean change in total 24-h, daytime, and nighttime BP after aerobic and combined (*n* = 19, the same participants), resistance (*n* = 23), and isometric handgrip (*n* = 18) exercise sessions, and the corresponding non-exercising control sessions were assessed in the original trials. The basic characteristics of the included studies as well as their timelines are described in Figure 1.

#### 2.2.1 Aerobic exercise

(Ferrari et al., 2017) The protocol was performed on a treadmill for 45 min at an intensity corresponding to 65%–70% VO_2max_, monitored through reserve heart rate or Borg rating of perceived exertion equivalent (i.e., Borg scale 11–13)18 for patients taking beta-blockers. Heart rate was monitored throughout the exercise session to ensure that exercise intensity was maintained.

#### 2.2.1 Combined exercise

(Ferrari et al., 2017) The protocol consisted of 20 min of resistance exercises followed by 25 min of aerobic exercise at 65%–70% VO_2max_. The resistance exercise protocol included four sets of eight repetitions per set, performed at 70% of the one-repetition maximum in the following sequence: bench press, bilateral knee extensors, bilateral elbow flexors, and bilateral knee flexors. An active interval of 2 minutes was allowed between sets for each exercise (i.e., exercises were grouped in a block of two, and within each block, the sets of the second exercise were performed during the rest of the first). Each contraction (concentric and eccentric) lasted 1.5 s and was controlled by an electronic metronome.

#### 2.2.3 Resistance exercise

(Schimitt et al., 2020) The protocol was composed of three sets of ten repetitions of five exercises performed in the following order: leg press, bench press, knee extension, upright row, and knee flexion, totaling approximately 40 min of exercise. Each exercise was performed at an intensity corresponding to 50% of one-repetition maximum and 2-min intervals between sets and exercises. During each repetition, the concentric phase of exercises was performed as fast as possible, while the eccentric phase lasted one to 2 seconds.

#### 2.2.4 Isometric handgrip exercise

(Bertoletti et al., 2022) The protocol comprised unilateral isometric handgrip exercise performed with the non-dominant hand. Four sets of 2 minutes each, with a 1-min rest between sets, totaling approximately 12 min of exercise. During each set, participants were asked to maintain approximately 30% of maximal voluntary contraction, and verbal feedback was provided during the exercise to maintain the intensity of the handgrip. Participants remained seated, feet completely flat on the floor, the back and forearm supported on the back and arm of the chair (respectively), the wrist in the neutral and free support position, the elbow flexed at 90°, and the shoulder slightly adducted and in the neutral position**.**

### 2.3 Classification of responders and non-responders

The inter-individual variability of the participants on post-exercise hypotension response was calculated using the net values of ABPM (exercise session minus control session). To categorize the participants as Responders and Non-responders, we designated the clinically meaningful change of reduction in systolic BP (4 mmHg) or diastolic BP (2 mmHg) (Liu et al., 2012). Participants were categorized as Responders for blood pressure when there was an improvement equal to or greater than 4 and 2 mmHg for systolic and diastolic BP, respectively; participants who scored below a clinically significant value were categorized as Non-responders.

### 2.4 Statistical analyses

The assumption of normality was analyzed using the Shapiro-Wilk test. Results were expressed as means, standard deviation and 95% confidence interval (Tables 1–4) for variables with normal distribution. The proportion of participants with post-exercise hypotension was calculated, and independent t-test analyses were performed to compare the clinical characteristics between Responders and Non-responders. For antihypertensive medication, the chi-square test was chosen to verify the association between use of antihypertensive medication and Responders/Non-responders when the expected frequency in each cell of the contingency table was greater than 5, and Fisher’s exact test when the contingency table was lower than 5. Statistical significance was set at *p* < 0.05. All statistical analyses were performed using SPSS Statistics for Windows version 22.0 (IBM, Armonk, NY, United States).

**TABLE 1:** Differences in characteristics between responders (>4 mmHg for systolic BP) and non-responders who did not present this response.

	Responders (*n* = 19)	Non-responders (*n* = 41)	Δ	*p*-value
**Anthropometry**				
Age, years	66 ± 3 (64 to 67)	66 ± 4 (64 to 68)	0 ± 8 (−2 to 3)	0.714
Body weight, kg	82 ± 10 (78 to 87)	77 ± 14 (73 to 82)	5 ± 30 (−12 to 2)	0.164
Height, cm	168 ± 7 (164 to 172)	166 ± 8 (162 to 169)	2 ± 8 (−8 to 3)	0.305
BMI, kg/m^2^	29 ± 3 (28 to 30)	28 ± 4 (27 to 29)	1 ± 8 (−3 to 1)	0.327
**Anti-hypertensive medications**	2 ± 1 (1 to 2)	2 ± 1 (1 to 2)	0 ± 8 (−1 to 1)	0.698
Diuretics, n (%)	9 (47)	26 (63)	—	0.625
β blockers, n (%)	3 (16)	9 (22)	—	0.565
Angiotensin converting enzyme inibitors, n (%)	5 (26)	12 (29)	—	0.436
Angiotensin receptor antagonists, n (%)	4 (21)	13 (32)	—	0.398
Calcium channel blockers, n (%)	6 (32)	9 (22)	—	0.276
Angiotensin II receptor blockers, n (%)	1 (5)	5 (26)	—	0.431
**Hemodynamic measures**				
Office systolic BP, mmHg	134 ± 16 (123 to 146)	139 ± 17 (133 to 145)	−5 ± 38 (−8 to 17)	0.451
Office diastolic BP, mmHg	80 ± 10 (73 to 87)	80 ± 11 (76 to 84)	0 ± 25 (−8 to 8)	0.998
Resting heart rate, bpm	66 ± 11 (60 to 72)	65 ± 11 (61 to 70)	1 ± 25 (−8 to 6)	0.791
**Systolic ABPM, mmHg**				
24-hour Exercise	131 ± 13 (125 to 138)	130 ± 11 (127 to 134)	1 ± 19 (−8 to 6)	0.768
24-hour Control	137 ± 13 (131 to 143)	128 ± 12 (125 to 132)	9 ± 19 (−15 to −2)	**0.018**
Δ 24-hour	−6 ± 4 (−9 to −4)	2 ± 6 (0 to 4)	−8 ± 15 (−12 to −5)	**<0.001**
Day-time Exercise	135 ± 14 (129 to 143)	134 ± 12 (130 to 137)	1 ± 19 (−9 to 5)	0.572
Day-time Control	140 ± 13 (134 to 147)	131 ± 13 (127 to 135)	9 ± 25 (−16 to −2)	**0.017**
Δ Day-time	−5 ± 5 (−7 to −3)	3 ± 6 (1 to 5)	−8 ± 15 (−11 to −5)	**<0.001**
Night-time Exercise	124 ± 15 (117 to 131)	121 ± 14 (116 to 125)	3 ± 25 (−11 to 5)	0.572
Night-time Control	129 ± 16 (121 to 136)	119 ± 14 (114 to 123)	10 ± 25 (−18 to −2)	**0.018**
Δ Night-time	−5 ± 14 (−14 to −1)	2 ± 8 (−1 to 4)	−7 ± 19 (−14 to −3)	**0.003**
**Diastolic ABPM, mmHg**				
24-hour Exercise	77 ± 8 (73 to 81)	78 ± 9 (75 to 80)	−1 ± 15 (−5 to 5)	0.837
24-hour Control	81 ± 8 (77 to 85)	77 ± 9 (74 to 79)	4 ± 15 (−9 to 1)	0.061
Δ 24-hour	−4 ± 4 (−6 to −2)	1 ± 3 (0 to 2)	−5 ± 8 (−6 to -3)	**<0.001**
Day-time Exercise	81 ± 7 (78 to 85)	80 ± 9 (77 to 83)	1 ± 15 (−6 to 4)	0.596
Day-time Control	83 ± 8 (80 to 88)	80 ± 10 (76 to 82)	3 ± 8 (−10 to 0)	0.059
Δ Day-time	−2 ± 5 (−5 to −1)	0 ± 3 (−1 to 1)	−2 ± 8 (−5 to −1)	**0.008**
Night-time Exercise	70 ± 10 (65 to 75)	70 ± 10 (66 to 73)	0 ± 19 (-6 to 5)	0.946
Night-time Control	74 ± 8 (70 to 78)	69 ± 10 (66 to 72)	5 ± 19 (−10 to 0)	0.065
Δ Night-time	−4 ± 7 (−8 to −1)	1 ± 4 (−1 to 2)	−5 ± 15 (−8 to −2)	**0.002**

Values are Means ± standard deviation (95% Confidence Interval); BMI, body mass index; ABPM, ambulatory blood pressure monitoring; Δ ABPM is comparing the results of the interventions (exercise session minus control session).

Bold *p*-values indicate significant results (*p* < 0.05).

## 3 Results

This pooled analysis resulted in 60 participants from three exercise training studies. The mean ± standard deviation of age was 66 ± 4 years, body weight 79 ± 13 kg, and body mass index 29 ± 3 kg/m^2^. The patients took 2 ± 1 antihypertensive medication [diuretics: *n* = 35 (58%); β blockers: *n* = 12 (20%), angiotensin converting enzyme inhibitor: *n* = 17 (28%), angiotensin receptor antagonists: *n* = 17 (22%), calcium channel blockers: *n* = 15 (25%), and angiotensin II receptor blockers: *n* = 6 (10%)] and had well-controlled office BP (129 ± 12 mmHg systolic BP and 75 ± 10 mmHg diastolic BP).

**FIGURE 2:**
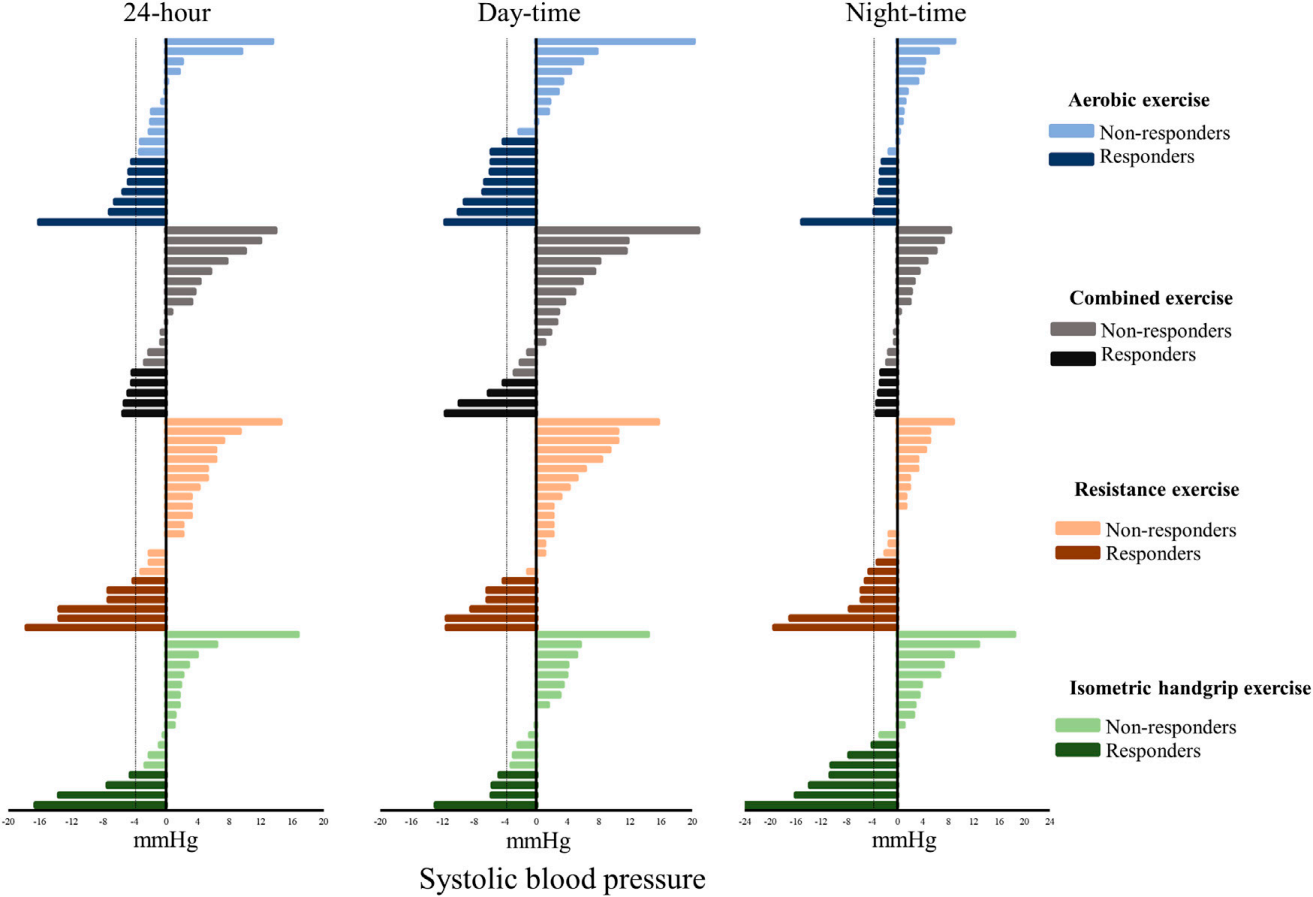
Individual changes in systolic blood pressure (exercise session minus control session). Dashed line: Minimal detectable change (4 mmHg).

**FIGURE 3:**
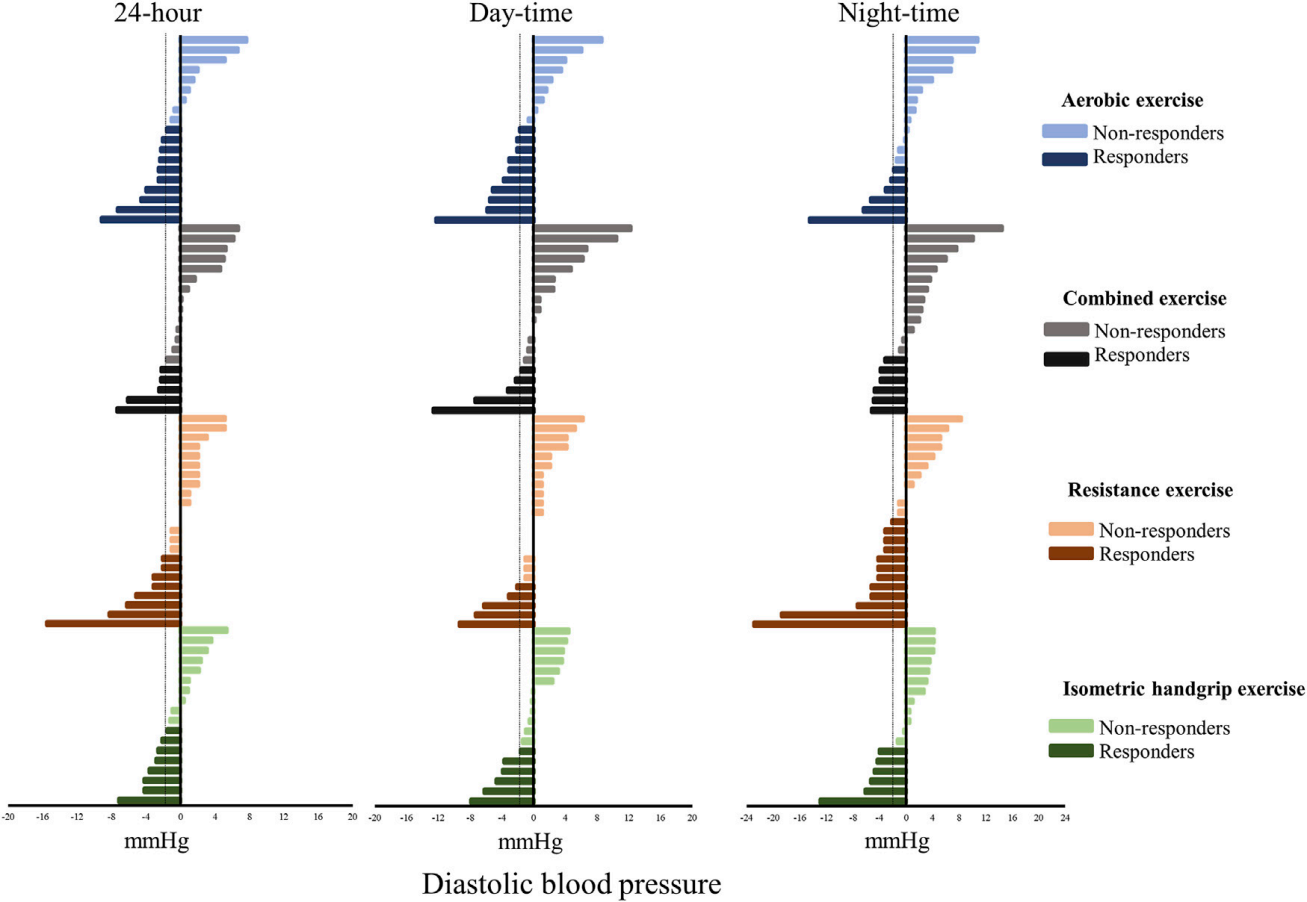
Individual changes in diastolic blood pressure (exercise session minus control session). Dashed line: Minimal detectable change (2 mmHg).

The Responders and Non-responders to various exercise modalities compared with control sessions are shown in Figures 2, 3. For systolic BP, the percentage of Responders was as follows: aerobic 24-h: 37%, daytime: 47% and nighttime: 37%; combined 24-h: 26%, daytime: 21% and nighttime: 32%; resistance 24-h: 26%, daytime: 26% and nighttime: 35%; and isometric handgrip 24-h: 22%, daytime: 22% and nighttime: 39%. For diastolic BP, the percentage of Responders was as follows: aerobic 24-h: 53%, daytime: 53% and nighttime: 31%; combined 24-h: 26%, daytime: 26% and nighttime: 31%; resistance 24-h: 35%, daytime: 22% and nighttime: 52%; and isometric handgrip 24-h: 44%, daytime: 33% and nighttime: 33%.

Table 1 shows the clinical characteristics of participants who presented a reduction of systolic BP > 4 mmHg (Responders) compared with Non-responders, and Table 2 presents the same comparisons for those who presented reductions of diastolic BP > 2 mmHg (Responders) compared with Non-responders. No significant differences were found for the use of anti-hypertensive medications between Responders and Non-responders.

**TABLE 2:** Differences in characteristics between responders (>2 mmHg for diastolic BP) and non-responders who did not present this response.

	Responders (*n* = 26)	Non-responders (*n* = 34)	Δ	*p*-value
**Anthropometry**				
Age, years	65 ± 3 (64 to 67)	66 ± 4 (65 to 68)	−1 ± 5 (−1 to 3)	0.445
Body weight, kg	81 ± 10 (77 to 85)	77 ± 15 (72 to 83)	4 ± 17 (−10 to 3)	0.329
Height, cm	167 ± 8 (162 to 170)	166 ± 8 (163 to 170)	1 ± 11 (−5 to 5)	0.305
BMI, kg/m^2^	29 ± 3 (28 to 30)	28 ± 4 (27 to 29)	1 ± 5 (−3 to 1)	0.327
**Anti-hypertensive medications**	2 ± 1 (2 to 3)	2 ± 1 (2 to 3)	0 ± 5 (−1 to 1)	0.698
Diuretics, n (%)	13 (50)	22 (65)	—	0.842
β blockers, n (%)	2 (7)	10 (29)	—	0.088
Angiotensin converting enzyme inibitors, n (%)	8 (30)	9 (26)	—	0.485
Angiotensin receptor antagonists, n (%)	6 (23)	11 (32)	—	0.907
Calcium channel blockers, n (%)	4 (15)	11 (32)	—	0.121
Angiotensin II receptor blockers, n (%)	4 (15)	2 (6)	—	0.417
**Hemodynamic measures**				
Office systolic BP, mmHg	136 ± 18 (126 to 147)	138 ± 15 (132 to 144)	−2 ± 29 (−9 to 13)	0.740
Office diastolic BP, mmHg	79 ± 12 (73 to 86)	80 ± 10 (76 to 84)	−1 ± 17 (−6 to 7)	0.887
Resting heart rate, bpm	67 ± 11 (62 to 72)	65 ± 11 (60 to 69)	2 ± 17 (−9 to 5)	0.522
**Systolic ABPM, mmHg**				
24-hour Exercise	128 ± 12 (124 to 133)	133 ± 11 (129 to 137)	−5 ± 17 (−2 to 10)	0.145
24-hour Control	131 ± 4 (126 to 137)	131 ± 12 (126 to 135)	0 ± 17 (−7 to 6)	0.852
Δ 24-hour	−3 ± 7 (−7 to −2)	2 ± 6 (0 to 4)	−5 ± 11 (−11 to −4)	**<0.001**
Day-time Exercise	132 ± 13 (127 to 138)	137 ± 12 (132 to 140)	−5 ± 17 (−10 to 2)	0.206
Day-time Control	135 ± 13 (130 to 141)	134 ± 14 (129 to 139)	1 ± 23 (−6 to 8)	0.709
Δ Day-time	−3 ± 6 (−6 to −1)	3 ± 6 (1 to 5)	−6 ± 11 (−10 to −3)	**<0.001**
Night-time Exercise	121 ± 14 (115 to 126)	123 ± 15 (117 to 128)	−2 ± 17 (−9 to 5)	0.535
Night-time Control	123 ± 17 (116 to 129)	121 ± 14 (117 to 127)	2 ± 23 (−7 to 8)	0.856
Δ Night-time	−2 ± 12 (−10 to −1)	2 ± 8 (−1 to 5)	−4 ± 17 (−12 to −2)	**0.006**
**Diastolic ABPM, mmHg**				
24-hour Exercise	75 ± 8 (73 to 79)	79 ± 10 (75 to 82)	−4 ± 11 (−7 to 1)	0.176
24-hour Control	78 ± 8 (75 to 81)	78 ± 9 (74 to 81)	0 ± 11 (−4 to 5)	0.782
Δ 24-hour	−3 ± 4 (−5 to −1)	1 ± 3 (0 to 2)	−4 ± 5 (−6 to −2)	**<0.001**
Day-time Exercise	79 ± 7 (76 to 82)	81 ± 10 (77 to 84)	−2 ± 11 (−6 to 3)	0.448
Day-time Control	81 ± 8 (78 to 85)	80 ± 10 (76 to 83)	1 ± 5 (−3 to 6)	0.533
Δ Day-time	−2 ± 4 (−4 to −1)	1 ± 3 (0 to 2)	−3 ± 5 (-5 to −2)	**0.001**
Night-time Exercise	68 ± 9 (65 to 73)	71 ± 10 (67 to 74)	−3 ± 17 (−7 to 3)	0.413
Night-time Control	71 ± 9 (67 to 75)	70 ± 10 (67 to 73)	1 ± 17 (−4 to 5)	0.726
Δ Night-time	−3 ± 7 (−7 to −1)	1 ± 4 (0 to 3)	−4 ± 11 (−8 to −2)	**0.001**

Values are Means ± standard deviation (95% Confidence Interval); BMI, body mass index; ABPM, ambulatory blood pressure monitoring; Δ ABPM is comparing the results of the interventions (exercise session minus control session).

Bold *p*-values indicate significant results (*p* < 0.05).

Tables 3, 4 present the clinical characteristics of Responders and Non-responders in each exercise modality. No significant differences were found for the use of anti-hypertensive medications between Responders and Non-responders after aerobic, resistance, combined and handgrip exercises.

**TABLE 3:** Differences in characteristics between responders and non-responders for 24-h systolic BP in each exercise modality.

	Responders	Non-responders	Δ	*p*-value
**Aerobic exercise, n (%)**	7 (37)	12 (63)	—	—
**Anti-hypertensive medications, n (%)**				
Diuretics	2 (29)	4 (33)	—	0.405
β blockers	2 (29)	3 (25)	—	0.701
Angiotensin converting enzyme inibitors	2 (29)	3 (25)	—	0.701
Angiotensin receptor antagonists	0 (0)	2 (17)	—	0.156
Calcium channel blockers	4 (57)	3 (25)	—	0.515
**Systolic ABPM, mmHg**				
24-hour Exercise	126 ± 6 (123 to 132)	122 ± 10 (115 to 128)	4 ± 10 (−4 to 12)	0.345
24-hour Control	133 ± 7 (128 to 138)	120 ± 7 (114 to 126)	13 ± 7 (6 to 20)	**0.001**
Δ 24-hour	−7 ± 4 (−9 to −2)	2 ± 6 (-2 to 6)	−9 ± 5 (−12 to -2)	**0.008**
**Diastolic ABPM, mmHg**				
24-hour Exercise	77 ± 7 (72 to 82)	73 ± 10 (68 to 80)	4 ± 10 (−7 to 10)	0.737
24-hour Control	81 ± 8 (75 to 86)	72 ± 7 (67 to 77)	9 ± 7 (2 to 16)	**0.016**
Δ 24-hour	−4 ± 3 (−6 to −1)	1 ± 4 (−1 to 5)	−5 ± 5 (−9 to −2)	**0.004**
**Combined exercise, n (%)**	5 (26)	14 (74)	—	—
**Anti-hypertensive medications, n (%)**				
Diuretics	2 (40)	4 (29)	—	0.637
β blockers	1 (20)	4 (29)	—	0.709
Angiotensin converting enzyme inibitors	1 (20)	4 (29)	—	0.709
Angiotensin receptor antagonists	0 (0)	2 (14)	—	0.372
Calcium channel blockers	3 (60)	4 (29)	—	0.211
**Systolic ABPM, mmHg**				
24-hour Exercise day	129 ± 8 (116 to 142)	128 ± 10 (122 to 134)	1 ± 11 (−10 to 12)	0.871
24-hour Control day	132 ± 7 (121 to 146)	124 ± 10 (118 to 130)	8 ± 8 (−2 to 18)	0.121
Δ 24-hour	−4 ± 1 (−5 to −4)	3 ± 5 (0 to 6)	−7 ± 4 (−13 to −2)	**0.008**
**Diastolic ABPM, mmHg**				
24-hour Exercise	76 ± 7 (64 to 88)	77 ± 9 (71 to 82)	−1 ± 11 (−12 to 10)	0.796
24-hour Control	78 ± 4 (71 to 85)	75 ± 9 (70 to 81)	2 ± 8 (−7 to 12)	0.594
Δ 24-hour	−2 ± 3 (−6 to 2)	2 ± 3 (−1 to 3)	−4 ± 4 (−7 to 1)	0.081
**Resistance exercise, n (%)**	6 (26)	17 (74)	—	—
**Anti-hypertensive medications, n (%)**				
Diuretics	4 (67)	15 (82)	—	0.231
β blockers	1 (17)	6 (35)	—	0.394
Angiotensin converting enzyme inibitors	1 (17)	5 (29)	—	0.210
Angiotensin receptor antagonists	4 (67)	10 (59)	—	0.735
Calcium channel blockers	1 (17)	4 (24)	—	0.726
**Systolic ABPM, mmHg**				
24-hour Exercise	129 ± 12 (116 to 141)	132 ± 12 (125 to 138)	−3 ± 12 (−14 to 9)	0.683
24-hour Control	139 ± 14 (124 to 154)	128 ± 12 (122 to 134)	11 ± 14 (−1 to 24)	0.075
Δ 24-hour	−10 ± 5 (−15 to −5)	4 ± 4 (1 to 6)	−14 ± 4 (−18 to −9)	**<0.001**
**Diastolic ABPM, mmHg**				
24-hour Exercise	72 ± 8 (64 to 81)	77 ± 9 (72 to 82)	−5 ± 12 (−15 to 5)	0.317
24-hour Control	79 ± 10 (68 to 89)	76 ± 10 (71 to 81)	3 ± 12 (−8 to 13)	0.597
Δ 24-hour	−7 ± 5 (−11 to −2)	1 ± 2 (0 to 2)	−8 ± 2 (−10 to −5)	**<0.001**
**Isometric handgrip exercise, n (%)**	4 (22)	14 (78)	—	—
**Anti-hypertensive medications, n (%)**				
Diuretics	3 (75)	7 (50)	—	0.558
Angiotensin converting enzyme inibitors	2 (50)	4 (29)	—	0.596
Angiotensin receptor antagonists	0 (0)	1 (6)	—	1000
Calcium channel blockers	1 (25)	2 (14)	—	1000
Angiotensin II receptor blockers	1 (25)	5 (36)	—	0.683
**Systolic ABPM, mmHg**				
24-hour Exercise	121 ± 10 (108 to 151)	139 ± 13 (119 to 145)	−18 ± 12 (−31 to −4)	**0.013**
24-hour Control	132 ± 10 (128 to 149)	137 ± 15 (126 to 144)	−5 ± 14 (−19 to 10)	0.470
Δ 24-hour	−11 ± 6 (−19 to −2)	2 ± 5 (0 to 5)	−13 ± 6 (−19 to −7)	**<0.001**
**Diastolic ABPM, mmHg**				
24-hour Exercise	77 ± 9 (63 to 91)	82 ± 6 (79 to 85)	−5 ± 8 (−12 to 3)	0.212
24-hour Control	76 ± 8 (63 to 88)	83 ± 6 (80 to 86)	−7 ± 6 (−14 to 0)	0.052
Δ 24-hour	1 ± 4 (−5 to 7)	−1 ± 3 (−3 to 1)	2 ± 4 (−1 to 6)	0.200

Values are Means ± standard deviation (95% Confidence Interval); BMI, body mass index; ABPM, ambulatory blood pressure monitoring; Δ ABPM is comparing the results of the interventions (exercise session minus control session).

Bold *p*-values indicate significant results (*p* < 0.05).

**TABLE 4:** Differences in characteristics between responders and non-responders for 24-h diastolic BP in each exercise modality.

	Responders	Non-responders	Δ	*p*-value
**Aerobic exercise, n (%)**	10 (53)	9 (47)	—	—
**Anti-hypertensive medications, n (%)**				
Diuretics	5 (63)	5 (50)	—	0.664
Angiotensin converting enzyme inibitors	4 (50)	2 (20)	—	0.321
Angiotensin receptor antagonists	0 (0)	1 (10)	—	1000
Calcium channel blockers	0 (0)	3 (30)	—	0.216
Angiotensin II receptor blockers	4 (50)	2 (20)	—	0.321
**Systolic ABPM, mmHg**				
24-hour Exercise	126 ± 6 (120 to 131)	127 ± 10 (118 to 136)	−1 ± 12 (−11 to 7)	0.673
24-hour Control	131 ± 10 (123 to 136)	125 ± 12 (115 to 138)	6 ± 15 (−4 to 17)	0.188
Δ 24-hour	−5 ± 4 (−9 to 2)	2 ± 5 (−4 to 4)	−7 ± 6 (−12 to −2)	**0.013**
**Diastolic ABPM, mmHg**				
24-hour Exercise	74 ± 7 (70 to 79)	76 ± 8 (69 to 84)	−2 ± 9 (−11 to 5)	0.421
24-hour Control	78 ± 9 (71 to 82)	74 ± 8 (67 to 83)	4 ± 12 (−3 to 12)	0.219
Δ 24-hour	−4 ± 2 (−6 to −2)	2 ± 3 (−1 to 5)	−6 ± 3 (−9 to −3)	**<0.001**
**Combined exercise, n (%)**	5 (26)	14 (74)	—	—
**Anti-hypertensive medications, n (%)**				
Diuretics	3 (60)	3 (21)	—	0.262
β blockers	1 (20)	4 (29)	—	0.709
Angiotensin converting enzyme inibitors	1 (20)	4 (29)	—	0.709
Angiotensin receptor antagonists	1 (20)	1 (7)	—	0.421
Calcium channel blockers	1 (20)	6 (43)	—	0.603
**Systolic ABPM, mmHg**				
24-hour Exercise	121 ± 3 (117 to 126)	130 ± 10 (124 to 136)	−9 ± 11 (−20 to 1)	0.076
24-hour Control	123 ± 5 (115 to 131)	128 ± 11 (121 to 134)	−5 ± 11 (−15 to 7)	0.470
Δ 24-hour	−2 ± 4 (−8 to 5)	2 ± 6 (-1 to 6)	−4 ± 6 (−11 to 2)	0.191
**Diastolic ABPM, mmHg**				
24-hour Exercise	69 ± 4 (62 to 75)	79 ± 8 (74 to 84)	−10 ± 8 (−20 to −1)	**0.028**
24-hour Control	72 ± 4 (66 to 78)	77 ± 9 (72 to 83)	−5 ± 8 (−14 to 5)	0.337
Δ 24-hour	−3 ± 2 (−6 to −1)	2 ± 3 (0 to 3)	−5 ± 2 (−8 to −2)	**0.005**
**Resistance exercise, n (%)**	8 (35)	15 (65)	—	—
**Anti-hypertensive medications, n (%)**				
Diuretics	6 (75)	13 (87)	—	0.589
β blockers	1 (13)	6 (40)	—	0.345
Angiotensin converting enzyme inibitors	3 (38)	4 (27)	—	0.709
Angiotensin receptor antagonists	5 (63)	9 (60)	—	0.907
Calcium channel blockers	2 (25)	3 (20)	—	0.787
**Systolic ABPM, mmHg**				
24-hour Exercise	128 ± 11 (119 to 138)	132 ± 12 (125 to 139)	−4 ± 14 (−15 to 7)	0.441
24-hour Control	136 ± 14 (124 to 148)	128 ± 13 (121 to 135)	8 ± 16 (−4 to 20)	0.171
Δ 24-hour	−8 ± 6 (−13 to −3)	4 ± 4 (2 to 7)	−12 ± 5 (−17 to −8)	**<0.001**
**Diastolic ABPM, mmHg**				
24-hour Exercise	72 ± 8 (66 to 78)	78 ± 11 (72 to 84)	−6 ± 11 (−15 to 3)	0.194
24-hour Control	78 ± 9 (70 to 85)	76 ± 10 (70 to 82)	2 ± 14 (−8 to 10)	0.798
Δ 24-hour	−6 ± 4 (−9 to −2)	2 ± 2 (0 to 3)	−8 ± 2 (−10 to −5)	**<0.001**
**Isometric handgrip exercise, n (%)**	8 (44)	10 (56)	—	—
**Anti-hypertensive medications, n (%)**				
Diuretics	5 (63)	5 (50)	—	0.664
Angiotensin converting enzyme inhibitors	4 (50)	2 (20)	—	0.321
Angiotensin receptor antagonists	0 (0)	1 (10)	—	1.000
Calcium channel blockers	0 (0)	3 (30)	—	0.216
**Systolic ABPM, mmHg**				
24-hour Exercise	134 ± 13 (123 to 145)	137 ± 12 (129 to 145)	−3 ± 19 (−18 to 10)	0.531
24-hour Control	133 ± 14 (121 to 145)	137 ± 14 (128 to 147)	−4 ± 16 (−15 to 10)	0.652
Δ 24-hour	1 ± 7 (−6 to 5)	0 ± 8 (−6 to 6)	1 ± 11 (−9 to 6)	0.741
**Diastolic ABPM, mmHg**				
24-hour Exercise	79 ± 4 (76 to 82)	83 ± 8 (77 to 88)	−4 ± 8 (−11 to 3)	0.217
24-hour Control	82 ± 4 (79 to 86)	81 ± 8 (75 to 87)	1 ± 8 (−6 to 8)	0.709
Δ 24-hour	−3 ± 2 (−5 to −2)	2 ± 2 (0 to 3)	−5 ± 2 (−7 to −3)	**<0.001**

Values are Means ± standard deviation (95% Confidence Interval); BMI, body mass index; ABPM, ambulatory blood pressure monitoring; Δ ABPM is comparing the results of the interventions (exercise session minus control session).

Bold *p*-values indicate significant results (*p* < 0.05).

## 4 Discussion

To our knowledge, no previous studies evaluated inter-individual variation of 24-h BP after a single bout of various exercise modalities in older adults with essential hypertension. A highlight of this study was that 37% of participants were Responders for 24-h systolic BP after aerobic exercise, 26% after resistance and combined exercises, and 22% following a single bout of isometric handgrip exercise. Assessing the general effects of exercise versus a control day without exercise revealed that 19 of 60 participants (32%) were Responders for systolic BP, and 24 of 60 participants (40%) were Responders for diastolic BP. The high baseline BP of Responders (−8 mmHg higher systolic BP on a day without exercise) suggests that this group might have the most significant capacity to decrease BP with various exercise modalities. The Responders also showed decreased systolic (−6 mmHg) and diastolic BP (−4 mmHg) after exercise compared to a non-exercising day. This result agrees with a network meta-analysis of 391 RCTs assessing exercise and BP-lowering medication that showed that participants with higher baseline values had more significant BP reduction after various exercise modalities (Naci et al., 2019).

We also found differences in the frequency of Responders among the exercise modalities. Although no previous study has assessed the inter-individual variation of 24-h BP after a single bout of different exercise modalities in older adults with hypertension, one study evaluated the inter-individual BP responses in patients with peripheral artery disease (*n* = 13; −65 years) after an aerobic exercise session (ten bouts of 2-min walking on a treadmill, interpolated by 2-min passive rest intervals; the intensity was adjusted at the speed previously determined to induce claudication pain symptoms) and a resistance exercise session (two times ten repetitions of eight exercises with a workload of 5–7 on the OMNI resistance exercise scale) (Lima et al., 2015). In this former study, a reduction of 4 mmHg in 24-h systolic/diastolic BP was adopted to categorize the participants as Responders, and the prevalence of Responders after resistance exercise (46% for systolic BP and 38% for diastolic BP) was higher than after aerobic exercise (31% for systolic BP and 15% for diastolic BP) (Lima et al., 2015). A potential explanation for the differences in the Responders among the intervention groups might be that resistance exercise was performed at moderate/vigorous intensity, and aerobic exercise was performed until the appearance of claudication pain symptoms, which might occur at low intensities depending on the participant.

For 24-h diastolic BP, aerobic also showed more significant results with 53% of Responders, followed by isometric handgrip (44%), resistance (35%), and combined (26%). In the original trials that described the group average responses after exercise (Ferrari et al., 2017; Schimitt et al., 2020; Bertoletti et al., 2022), except for the results of aerobic exercise intervention (Ferrari et al., 2017), any exercise acutely reduced 24-h ambulatory BP. Because 24-h ABPM is the gold standard for measuring BP (Niiranen et al., 2014), and combined and resistance training are cornerstones for improving functionality in older adults (Fragala et al., 2019), this finding reinforces the notion that different exercise modalities might be advantageous for older adults with hypertension. Some potential physiological mechanisms underlying the inter-individual variability in post-exercise hypotension following different exercise modalities can be speculated. Aerobic exercise has a sympatholytic effect and decreases the alpha-adrenergic response to sympathetic stimuli, resulting in a reduction in peripheral vascular resistance (Halliwill et al., 1996), and may secrete some vasodilator substances such as nitric oxide, prostaglandins, histamine, which may contribute to the maintenance of vasodilation after exercise, inducing a greater PEH in this population (Halliwill et al., 2013).

Some limitations of the present study should be considered. Although this original analysis provides insights for the design of future RCTs, it broke the randomization of the primary studies, and the sample size was not calculated to compare Responders vs. Non-responders. The enrollment of untrained participants aged 60–75 years might have limited the generalizability of our findings to younger or trained participants; however, analyses of functioning and untrained older adults are more likely to represent the general elderly population. The strength of this analysis was that it provided pooled individual data from participants of three RCTs using the same standardized methodology (e.g., researchers who allocated the data and those who analyzed it were blinded to the outcome; all experiments started at the same time of day to control for a potential diurnal variation on BP and residual effects of antihypertensive medications, and all participants received recommendations to maintain the same routine throughout the study). We used the same equipment to perform ABPM (Spacelabs model 90207), the gold standard for understanding BP behavior during the day and sleep. Furthermore, the study included subjects with similar characteristics (e.g., older adults with hypertension, sedentary, living in the same city), allowing a comprehensive exploration of the inter-individual variation of BP following different exercise modalities.

## 5 Conclusion

Older adults with hypertension present high inter-individual variability of BP after a single bout of various exercise modalities. Twenty-four-hour post-exercise hypotension occurred in participants with higher BP values on the control day without exercise, and different exercise modalities might effectively reduce 24-h BP in older adults with hypertension acutely. For the Non-responders, personalized exercise interventions can play a critical role in reducing BP, whereas those with low sensitivity to a generalized exercise prescription might require more significant exercise stimuli (i.e., volume, intensity).

## Data Availability

The raw data supporting the conclusions of this article will be made available by the authors, without undue reservation.
